# Does transport affect the eating quality potential of beef from Limousin cows in France? - A case study

**DOI:** 10.1016/j.vas.2024.100411

**Published:** 2024-11-12

**Authors:** Nathalia da Silva Rodrigues Mendes, Renato Rodrigues Silva, Moïse Kombolo-Ngah, Pierre-Philippe Rivet, Jerôme Tondusson, Tatianne Ferreira de Oliveira, Sghaier Chriki, Marie-Pierre Ellies-Oury, Jean-François Hocquette

**Affiliations:** a Institut National de Recherche pour l'Agriculture, l'Alimentation et l'Environnement (INRAE), Université Clermont Auvergne, VetAgro Sup, UMR1213, Recherches sur les Herbivores, Theix, 63122 Saint-Genès Champanelle, France; b Federal University of Goiás - UFG, Campus Samambaia, Rodovia Goiânia-Nova Veneza Km-0, Caixa Postal 131, CEP 74690-900, Goiânia, Brazil; c CV Plainemaison, Av. De L'Abattoir, 87000 Limoges, France; d ISARA, 23 rue Jean Baldassini, CEDEX 07, 69364 Lyon, France; e Bordeaux Sciences Agro, CS 40201, 33175 Gradignan, France

**Keywords:** Pre-slaughter stress, Animal welfare, Beef eating quality, MSA, Carcass traits

## Abstract

We hypothesized that transportation might impact beef sensory quality, particularly as the distance between farms and the slaughterhouse increases. Specifically, we expected that pre-slaughter transport over longer distances would induce stress in the cattle, resulting in elevated ultimate pH and, consequently, reduced beef quality. Thus, this study aimed to specifically study the effects of transport in commercial conditions of Limousin cows from farms located in different areas of France to slaughter on ultimate pH (pHu) measured in the *Longissimus thoracis* muscle (LT), marbling (two major factors affecting eating quality) and the MSA Index (a global indicator of potential eating quality at the carcass level). Cattle were studied according to distance (<50 km; between 50 and 150 km; and between 150 and 250 km from the slaughterhouse) and according to transport time from the farm to the slaughterhouse (divided into short, moderate, and extended). We found that neither distance nor transport time significantly affected pHu of beef and the other studied parameters (cold carcass weight, meat color, MSA Marbling and MSA Index). Consequently, in our commercial conditions, we concluded that the effects of transport on beef carcasses and beef eating quality of Limousin cows are negligible.

## Highlights

• Effects of transport on the sensory quality potential of Limousin beef was investigated.
• Transport time did not affect pHu, marbling, carcass weight or MSA index.
• Effects of transport on beef eating quality potential were negligible.

## 1 Introduction

The effect of road transport on beef quality is a multifaceted issue encompassing various stressors such as load density, microclimates, handling procedures, conditions and duration of transport (González, Schwartzkopf-Genswein, Bryan, Silasi & Brown, 2012; Mendes et al., 2024b; Schwartzkopf-Genswein, Ahola, Edwards-Callaway, Hale & Paterson, 2012, 2016). These have been shown to have a negative effect on beef eating quality in addition to raising animal welfare concerns (Astruc & Terlouw, 2023; Gruber et al., 2010; Padalino, Menchetti, Mininni, Tullio & Nanni Costa, 2021; Prache et al., 2022; Wigham, Butterworth & Wotton, 2018). Transport time appears to have the most pronounced consequences on beef quality with significant economic impacts because of its effects on carcass weight, sensory qualities, and mortality rates during transport (González et al., 2012).

In order to reduce these stress factors associated with animal transport, European Commission (EC) (2005) was promulgated. This regulation imposes specific requirements depending on transport duration (whether less than or greater than 8 h) and the species transported (specifying minimum load density and maximum duration of the journey). The European Food Safety Authority (EFSA) (2022) recently stipulated that adult cattle should not be transported for longer than 29 h, after which they must have access to food and water for 24 h.

Ultimate pH (pHu) is a relevant indicator of beef quality, significantly influencing its shelf life, processability, and water retention, and can be used to infer the effects of transport from farm to slaughterhouse on the final quality of beef. Of particular importance are the effects of suboptimal handling during transportation (Ponnampalam et al., 2017), which can result in muscle glycogen depletion, inadequate acidification, and less than optimal ultimate pH after slaughter (Gagaoua et al., 2021). This can lead to darker beef with less desirable sensory properties, especially reduced tenderness, juiciness, and flavor (Gruber et al., 2010), resulting in a higher incidence of DFD (Dark, Firm and Dry) beef (Gruber et al., 2010; Hemsworth et al., 2011). Insufficient glycogen stores result in beef exhibiting characteristics associated with DFD (Ponnampalam et al., 2017; Wicks et al., 2019). Since each animal reacts to stress differently, stress levels prior to slaughter need either to be directly measured or to be kept at a very low level. Due to this variability of stress reactions to environmental stimuli, statistical models used to predict beef eating quality need to take this variability into account (Terlouw et al., 2021). In practice, dark-cut carcasses are discounted during beef carcass grading and devalued, which causes economic losses for the beef sector. This is the case, for example, when using grading schemes such as Meat Standards Australia (MSA) where carcasses with pHu> 5.7 and meat color scores >3 are excluded.

A challenge in this context is to correctly assess the sensory quality of beef. So far, the best approach to do so is the MSA grading scheme (Bonny et al., 2018b; Mendes et al., 2024a). It is based on precise protocols to collect data gathered both before and at the slaughterhouse to predict beef eating quality (Bonny et al., 2018b). MSA is regarded as one of the most sophisticated beef grading systems, because it not only evaluates the potential eating quality of various cuts according to different cooking methods (Bonny et al., 2018b), but also estimates the potential eating quality of the whole carcass (McGilchrist, Polkinghorne, Ball & Thompson, 2019)**.** A European adaptation of the MSA protocol which is the 3G (Guaranteed Global Grading), (Global Guaranteed Grading 2023) protocol is being studied in different European countries (Hocquette et al., 2020).

Information on the effect of transport time on beef eating quality in practical and commercial conditions is still limited, especially in France, the leading beef producer in the European Union (EUROSTAT, 2022; reviewed by Mendes et al., 2024a). Indeed, only a few studies have had access to large commercial datasets to investigate the effects of stress during transport, with the notable exceptions of Levakhin et al. (2017) with calves from the Russian federation, Polkinghorne, Philpott and Thompson (2018) with Australian steers and Mendes et al. (2024b) with mainly entire males from Brazil. Mendes et al. (2024b) concluded that long distances did not have a significant impact on ultimate pH and therefore on quality potential of beef from Nellore males in Brazil. Similarly, Polkinghorne et al. (2018) showed that transportation had no significant effect on characteristics of live steers and carcasses, consumer sensory scores and objective beef quality of the *Longissimus lumborum*, also known as striploin. Levakhin et al. (2017) indicated a higher sensitivity to transport of Limousin calves compared to other cattle types.

In addition, predicting beef eating quality using commercial data is a challenge except when using the MSA model when carcasses are graded according to the MSA protocols. The only study on transport effect on consumer sensory scores using the MSA grading scheme is that of Polkinghorne et al. (2018) with Australian steers, but this work was limited to effects on eating quality potential of the striploin.

The MSA grading scheme is based on different predictors of eating quality including marbling, known to be positively correlated with beef sensory traits such as juiciness, color, tenderness, and taste (Stewart et al., 2021). The French National Food Conference recently recommended that the meat sector, represented by INTERBEV, should introduce marbling into the French beef classification system (EtatsGénéraux de l'Alimentation EGA, 2018). The inclusion of marbling in the French or even European classification scheme could improve the quality and economic value of beef products as there is no significant relationship between the EUROP carcass classification and the eating quality of beef (Bonny et al., 2016, Bonny et al., 2017; Liu et al., 2020). Moreover, the MSA index is another crucial parameter of interest recently suggested to assess the potential eating quality of the whole carcass (McGilchrist et al., 2019). This index describes the average consumer eating experience for the entire carcass by combining all the eating quality scores (MQ4), by weighting tenderness, juiciness, flavor liking and overall acceptability scores of each muscle based on its most common cooking method, MQ4 scores being weighted proportionally to the weight of each individual cut in relation to the total weight of all cuts (Bonny et al., 2018a).

Based on this updated tools and knowledge, for the first time in France, potential consequences of transport stress on beef eating quality were assessed in commercial conditions using the MSA index at the carcass level. Indeed, we hypothesized that transportation might impact beef sensory quality, particularly as the distance between farms and the slaughterhouse increases. Additionally, this study investigated for the first time the effects of stress of cows (which produce the major part of beef in France) from a late-maturing breed (Limousin) during transport to slaughter from farms located in different geographical areas, considering that the Limousin breed may be more sensitive to transport stress (Levakhin et al., 2017).

## 2 Material and methods

### 2.1 Data set

The dataset used in this study, provided by CV Plainemaison-Beauvallet, a commercial slaughterhouse in Limoges, consists of records from 4407 beef carcasses from Limousin cows, with each carcass having its own record. Quality checks were applied to validate this dataset, including verification of completeness (ensuring all records contained necessary information), consistency checks across different variables, and exclusion of records with missing or implausible values. These procedures were implemented to ensure the reliability and integrity of the data used in the analysis. This slaughterhouse is the major one involved in beef production from the Limousin breed. It was also selected to ensure that all animals were processed under consistent and standardized conditions, minimizing variability in carcass treatment and, thus, ensuring the reliability of the results. This choice was crucial for maintaining the integrity of the study's focus on the effects of transport, as it avoided potential confounding factors associated with differing slaughter practices. These cattle were slaughtered between January 2020 and October 2022. Entries contain among others beef carcass traits, such as age in months (itself related to animal maturity) and cold carcass weight (CCW). Carcass weight is typically measured within 2 h of slaughter after removal of head, hide, feet/legs, thoracic organs, internal fats, and internal organs and is expressed as cold carcass weight, which is 0.98 times the hot carcass weight according to the EUROP guidelines (Commission of the European Communities, 2008).

**Fig. 1:**
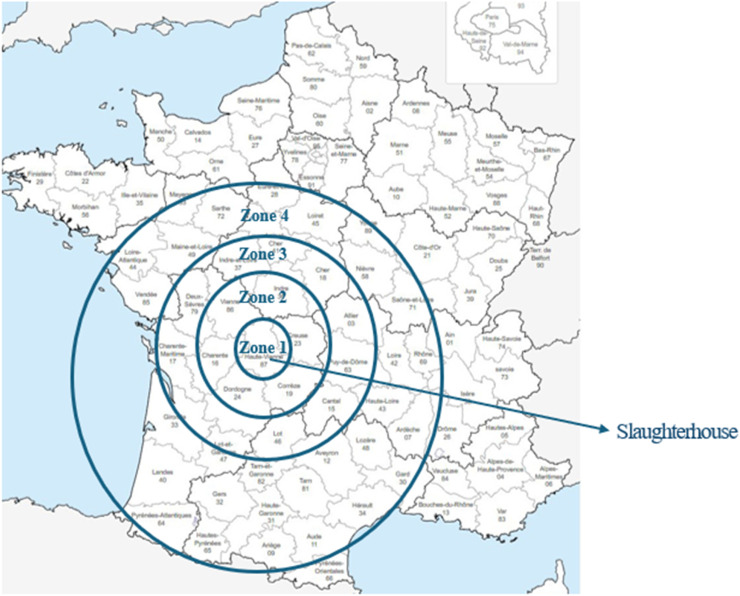
Geographical distribution of farms by distances from farms to the slaughterhouse.

**Fig. 2:**
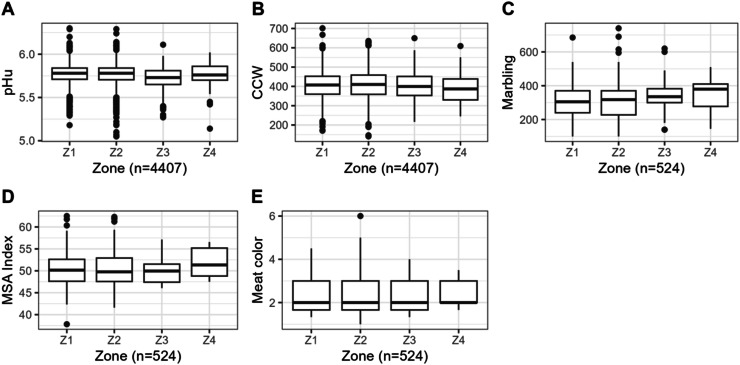
Box plots of the distribution of pHu (*n* = 4407 carcasses) (A), Cold Carcass Weight (CCW, *n* = 4407 carcasses) (B), Marbling (*n* = 524 carcasses) (C), MSA index (*n* = 524 carcasses) (D) and Meat color (E) (*n* = 524 carcasses) in the different geographical zones studied in France.

**Fig. 3:**
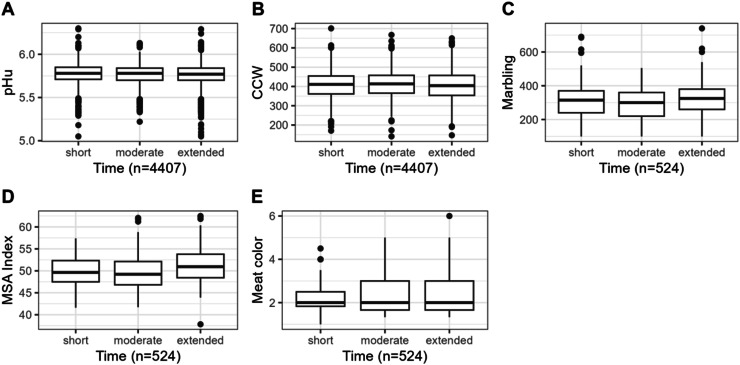
Box plots of the distribution of pHu (*n* = 4407 carcasses) (A), Cold Carcass Weight (CCW, *n* = 4407 carcasses) (B), Marbling (*n* = 524 carcasses) (C), and MSA index (*n* = 524 carcasses) (D) and Meat Color (E) according to transport time class (short, moderate and extended).

The company purchased these animals from different geographical areas in France, but mainly from the central-western region (Zones 1 and 2) because of their proximity to the slaughterhouse. These animals are representative of beef cattle raised in the Limousin region of France and all animals were transported by very experienced drivers. The drivers and stockyard staff had previously received training in animal welfare practices. The geographical zones were defined as follows: zone 1 (less than 50 km from the slaughterhouse), zone 2 (between 50 and 150 km), zone 3 (between 150 and 250 km) and zone 4 (>250 km), as shown in Fig. 1. Transport time from the farm was an average of 1.3 h (see Table 1). The transport time from farms to the slaughterhouse was divided into the following categories for pHu and CCW: short (less than or equal to 47 min), moderate (between 47 and 71 min) and extended (> 71 min). For MSA data, Marbling and MSA Index, these categories were short (less than or equal to 22 min), moderate (between 22 and 42 min) and extended (> 42 min). These divisions are derived from the box plots in Fig. 2, Fig. 3 (the box extends from the 25th to the 75th percentile, with whiskers indicating minimum and maximum values).

**Table 1:** Descriptive statistics (mean, standard deviation (SD), coefficient of variation (CV), minimum, and maximum) for the measured traits for the studied cattle population.

	n	Mean	SD		CV (%)	Minimum	Maximum
ultimate pH	4407	5.76		0.13	2.22	5.05	6.30
Distance (km)	4407	98.96		71.03	71.77	5.5	596
Time (min)	4407	78.87		46.39	58.82	12	364
Animal age (months)	4407	110.49		46.39	45.03	5	256
Cold Carcass Weight (kg)	4407	407.88		70.96	17.77	140.9	701.2
EU Conformation score 1	4407	8.93 (*R*+)		1.96	21.99	1	14
EU Fat score 2	4407	7.49 (3-)		2.83	37.77	1	9
Marbling	524	310.8		100.34	32.29	100	740
Meat Color	524	2,31		0.75	32.51	1 (1A)	6
MSA Index^3^	524	50.46		4.03	7.99	37.82	62.49

1 European conformation score were converted from P (- /=/+), O (- /=/+), R (- /=/+), U (- /=/+), and E (- /=/+) to classes from 1 (P-) to 15 (*E*+) according to Hickey et al. (2007).

2 European fat scores were converted from 1 (- /=/+), 2 (- /=/+), 3 (- /=/+), 4 (- /=/+), and 5 (- /=/+) to classes from 1 (1-) to 15 (5+) according to Hickey et al. (2007).

3 5-day MSA Index = carcass predicted MSA score calculated as the weighted sum of the predicted MQ4 (meat quality score) scores of all MSA cuts. The model assumes that all the animals were Achilles hung, and that all cuts were aged for 5 days and cooked according to the most common cooking method for each cut (McGilchrist et al., 2019).

Animals were handled in accordance with the French animal protection regulations defined by the French legislation (Code Rural, articles R214–64 to R214–71; Legifrance, 2016). Animals from zones 3 and 4 were transported to the slaughterhouse the day before slaughter. In all cases and for all zones, all animals were in good conditions on arrival. Before slaughter, all animals were not given food for 24 h but had unrestricted access to water. Exsanguination from the jugular vein was performed after use of an electrical captive-bolt pistol. Slaughter was performed in accordance with EU regulations (European Commission (EC), 2009). Carcasses were dressed according to standard commercial practices and were split between 30 and 50 min post exsanguination then chilled for 24 h at 2 – 4°C.

### 2.2 Measurements

The carcasses were assessed according to EUROP criteria. This was done at the slaughterhouse by experts of the EUROP grid (Commission Regulation (EC) 1249/2008). According to the EUROP grid, five conformation classes were used (E, U, R, O, and P) as well as five fatness classes (from 1 to 5). Assessments of carcass conformation fatness were based on visual inspection of carcasses. Depending on the degree of muscularity, carcasses received a score of ‘E’ for the most muscularity through to ‘P’ for carcasses with the least muscularity. European Union regulations have 3 subdivisions for each conformation: high “+”, medium “=” and low “−”. For this reason, an incremental scale ranging from 1 to 15 was used, with 1 corresponding to *P*− (very low muscularity) and 15 to *E*+ (very high muscularity). In addition, the degree of fat cover of the carcasses (hereafter fatness score), which corresponds to the amount of fat on the outside of the carcass, was numerically scored from 1 (the leanest) to 5 (the fattest), with 3 subdivisions for each fatness score (-, = and +) as for conformation. This conversion of European conformation and fat scores into a continuous 15-point scale has been described by Hickey et al. (2007).

Ultimate pH (pHu) was assessed after 24 h of *postmortem* chilling at an average temperature between 0 and 4°C. Before measurement, the pHu meter was first calibrated at chilling temperature using pH 4 and pH 7 buffers. The pHu was measured in the *Longissimus thoracis* muscle (LT) between the 6th and 7th ribs, anterior striploin piece.

### 2.3 MSA grading of carcasses and calculation of the MSA index

In addition, 524 carcasses were graded for MSA traits: ossification and marbling scores, rib fat thickness at the 10th rib and hump height using MSA chiller assessment standards by an MSA-certified grader under the auspices of AUS-MEAT and the International Meat Research 3G Foundation (AUS-MEAT, 2018).

After 24 h of *postmortem* chilling at an average temperature between 0 and 4°C, carcasses were graded. Marbling assessments were performed in accordance with the ABCAS reference standards (Meat, Livestock, Australia & Meat Standards, Australia, 2001,^2018^) as recently described (Kombolo-Ngah et al., 2024), following the UNECE Bovine Language Standards. The marbling standards have been tailored to European cattle and consumers through extensive collaborative research in Europe, with data storage facilitated by the IMR3GF (International Meat Research 3G Foundation).

The MSA marbling score, which provides a more detailed scale (ranging from 100 to 1190 in increments of 10), was used to indicate the amount, size, fineness and distribution of fat inclusions. Evaluation of MSA marbling scores and Meat color were performed at the 5th rib of the carcass according to the methodology described by Liu et al. (2021). A grader certified by the AUS-MEAT and International Research Meat 3G Foundation performed the assessments. The grader underwent uniform training and maintained certification in accordance with the Australian Beef Chiller Assessment System (ABCAS) standards in order to minimize variability in assessments. After cutting, the ribeye was exposed to air for a minimum of 20 min and up to 3 h to allow the meat to bloom prior to assessment according to MSA procedures. Grading was performed using standard visual cards provided by ABCAS to assess MSA scores.

The MSA index was developed to provide feedback to the producer about the potential eating quality of his beef carcasses, with the opportunity of ranking animals and monitoring the impact of management and genetic changes on eating quality (McGilchrist et al., 2019). The MSA index was calculated with assumption of the standard aging time of 5 days. More detailed information about the MSA grading can be found in McGilchrist et al. (2019).

### 2.4 Statistical analysis

The data were analyzed using R software (version 4.3.0 - R Core Team, 2023). Descriptive analysis was done using a box plot according to DuToit, Steyn and Stumpf (2012). Median values are indicated by the line within the box plot.

One way classification analysis of variance model (ANOVA) was performed on the data set using the aov() function to determine significant differences between treatments:

$$y_{\mathrm{ij}}=\mu +\tau_{i}+\epsilon_{\mathrm{ij}}\;;\;i=1....,\;a,\;j=1,...n.$$

where $y_{ij}$ represents the ij-th measured values for the respective response variable of jth observation in the ith treatment, μ is the intercept, $\tau_{i}$ is the treatment effect, and $\epsilon_{ij}N\left(0,\sigma^{2}\right)$ is the random error. In this model, the treatment represents either the geographic zone or the class of transport time, and the response variable represents the ultimate pH, Cold Carcass Weight (CCW), the Marbling score, Meat color or the MSA index. Tukey tests were performed to determine which means differ in a set of contrasts (Behrendt, 2014).

We also fitted a mixed model to determine the relationship between each response variable related to meat eating quality (ultimate pH (pHu), Cold Carcass Weight (CCW), MSA Marbling, and MSA index) and geographic zones in France or class of transport time, while accounting for the random effects of producers within each zone or class of transport time. Meat color is not a predictor of quality in the MSA Index and was therefore not included in the linear mixed model analysis. This approach was to confirm the previous ANOVA analysis especially when data were not normally distributed (i.e. with a significant Bartlett test for the variables ultimate pH and cold carcass weight in different times divided into short, moderate and extended). The statistical model can be expressed as follows:

$$y_{ij}=\mu +\tau_{i}+b_{ij}+\epsilon_{ij}$$

where $y_{ij}$ represents the i th measured values for the respective response variable for observation j in zone or class of transport time, μ is the intercept, $\tau_{i}$ is the fixed effect of geographic zone or class of transport time, $b_{ij}N\left(0,\sigma_{b}^{2}\right)$ is the random effect of the producer within the zone or class of transport time and $\epsilon_{ij}N\left(0,\sigma^{2}\right)$ is the random error. This model allowed us to assess the impact of class of transport time on pH while considering the variability introduced by individual producers within each zone or class of transport time (Pinheiro, Bates & Core Team, 2023).

## 3 Results and discussion

### 3.1 Variability in carcass data between and within geographical zones

Descriptive statistics of animal and carcass traits of Limousin cows are reported in Table 1.

The ultimate pH mean value for the current study was 5.76 ± 0.13, with a coefficient of variation of 2.22% (Table 1), which is consistent with findings from Liu et al. (2021), who reported mean values of 5.7 ± 0.16. Additionally, the mean pHu values reported in other studies are within the normal range for cattle, with values of 5.59 ± 0.14 and 5.57 ± 0.11 with or without extreme values, respectively (Gagaoua, Picard & Monteils, 2017, 2018a, 2018b). This indicates that most of the animals were not likely to have experienced significant pre-slaughter stress, since 3.43% of the cows had an ultimate pH higher than 6.0, which is a threshold associated with glycogen loss related to stress (Gagaoua, Picard, Soulat & Monteils, 2018c).

It should be noted that there is no technical or regulatory limit in France for meat with a high ultimate pH. Whereas the Afnor V46 001 standard (December 1996) refers to a value of 6.0, the generally accepted limit is between 5.8 and 6.0. This limit varies according to market requirements (INTERBEV, 2023). These results are generally consistent with MSA standards, reflecting acceptable levels of pHu for commercial grading. Additionally, strict adherence to MSA requirements is not necessary for research purposes, allowing for some flexibility in exploring different aspects of meat quality under varied conditions (MLA, personal communication).

It should be noted that a pH value exceeding 5.8 is frequently designated as DFD (Dark, Firm and Dry) by some researchers (Hughes, Clarke, Purslow & Warner, 2017; Lomiwes, Farouk, Wu & Young, 2014; Ponnampalam et al., 2017). Such variations in ultimate pH can lead to significant losses in the beef industry. It is worth noting that stress in cattle can result in weight loss, carcass lesions, and reduced meat quality. These are primarily due to elevated ultimate pH levels (>5.8), which can impact tenderness and meat color (resulting in darker meat) (Hughes et al., 2017; Lomiwes et al., 2014).

Only the pHu showed any significant zone-dependent variation (Table 2). These findings suggest that monitoring ultimate pH levels in carcasses post-slaughter and implementing strategies to minimize stress in cattle would ensure higher meat quality and reduce economic losses in the beef industry (Hughes et al., 2017; Ponnampalam et al., 2017).

**Table 2:** Carcass traits in different geographic zones in France of the following traits pHu (*n* = 4407 carcasses), Cold Carcass Weight (CCW, *n* = 4407 carcasses), Meat color and Marbling (*n* = 524 carcasses) and MSA Index (*n* = 524 carcasses).

	Geographic zone according to the distance between the farm and the slaughterhouse				SEM^1^	*P* value
	Zone 1	Zone 2	Zone 3	Zone 4		
pHu	5.77^a^	5.76^a^	5.72^b^	5.75^a^^,^^b^	0.002	<0.05
CCW (kg)	405.84	409.70	403.64	392.81	1.069	NS
Marbling	308.46	306.20	333.55	354.21	4.374	NS
Meat Color	2.30	2.31	2.43	2.31	0.030	NS
MSA Index	50.36	50.50	50.13	51.91	0.227	NS

a,b Means with different letters within a row are significantly different (p value <0.05).

1 SEM - Standard error of the mean.

The mean carcass weight was 407.88 ± 70.96 kg with a coefficient of variation of 17.77% (Table 1). This is consistent with findings by Liu et al. (2021), who reported a mean carcass weight of 356.5 ± 95.8 kg for predominantly Limousine cows. As shown in Table 2, no significant difference in cold carcass weight was observed across different zones.

The mean MSA marbling score obtained in our study (310.8) was slightly higher than that of 288 at the 5th rib reported by Liu et al. (2021). This result was derived from 208 mainly French Limousine cows, which were graded according to the Australian Beef Carcass Chiller Assessment System (ABCAS) used in the MSA/3G grading scheme. Carcasses analyzed in the current study were slightly heavier than those of Liu et al. (2021), which is consistent with more expressed marbling. Consistent with Liu et al. (2021) and this study, Polkinghorne et al. (2018) found an MSA Marbling score of 291. Santinello et al. (2024) evaluated a dataset comprising 55 young bulls and heifers from late-maturing breeds at an Italian abattoir. This assessment was performed in accordance with MSA guidelines, considering both the locations and sides of carcass grading. It reported a mean MSA marbling score of 458, which is notably higher than the marbling values obtained in our study and by Liu et al. (2021). This can be partly explained by the intensive rearing conditions in specialized Italian fattening farms, where young bulls are fed a diet rich in concentrates for six months before slaughter (Santinello et al., 2024).

The mean meat color score observed in our study (2.30) was slightly lower than those reported by Liu et al. (2021) at the 5th rib (2.5) in a study conducted in France, which used 208 mainly French Limousin cows, and significantly lower than the results found by Polkinghorne et al. (2018) at the 10th rib in a study conducted in Australia, which involved a total of 343 steers (3.2). Factors such as diet, ultimate pH, and muscle type can influence the characteristics of meat color (Mancini, Hunt, 2005), which explains the differences observed between the studies. Notably, there was no significant difference in meat color across different geographical regions within. The mean meat color in our study is considered acceptable according to the MSA grading scheme (<3), although meat color is not a parameter used in the prediction model of the MSA Index, as outlined by the MSA grading system (AUS-MEAT, 2018).

The mean MSA Index found in the current study (Table 1) was similar (50.46) to the values reported by Liu et al. (2021). However, it should be noted that the MSA marbling score plays a key role in determining the MSA Index, especially when other predictive factors are held constant (same hanging method, no *Bos indicus* content, no hormone growth promotion status, etc.) (Pethick, Hocquette, Scollan & Dunshea, 2021). Furthermore, Limousine cull cows are generally slaughtered once they have completed their growth phase, and therefore exhibit homogeneous marbling deposition along grading sites. This is likely related to their greater age (Liu et al., 2021). Santinello et al. (2024) observed a higher MSA index (61) for young bulls. This may be explained by the fact that the cull cows are likely producing less muscle rich in connective tissue than younger animals, which may decrease tenderness. In addition, young animals in the Italian finishing system are fed a diet rich in concentrates, which favors marbling deposition (Santinello et al., 2024) and therefore eating quality.

Mixed linear models were fitted to data, and confidence intervals for variance components and contrast of least square means were estimated. Results from the 95% confidence interval for the variance of components of the producer effect within zones the carcass traits within geographic zones showed that there is significant variability between producers in Cold Carcass Weight, Marbling and MSA Index, but pHu showed no significant variability between producers (Table 3).

**Table 3:** Lower and upper limits of the 95% confidence interval for the variance components of the producer effect within zones of the following traits pHu (*n* = 4407 carcasses), Cold Carcass Weight (CCW, *n* = 4407 carcasses), Marbling (*n* = 524 carcasses) and MSA Index (*n* = 524 carcasses).

	σ^2^	
Trait	2.5%	97.5%
pHu	0.00	0.05
Cold Carcass Weight (kg)	68.09	71.19
Marbling	31.92	59.47
MSA Index	1.65	3.03

Furthermore, when accounting for variability between producers, the contrasts of least square means revealed statistical significance only between zone 1 and 3, and between zone 2 and 3, regarding ultimate pH (pHu), as well as between zone 1 and 4 for marbling. Values of pHu were found to be lower in the geographic zone 3, far from the slaughterhouse (5.72 versus 5.77, *P* <0.05, Fig. 2). Importantly, the rest periods for cattle in zones 1 and 2, as well as in zones 3 and 4, were similar, with animals from zones 3 and 4 having been transported the day before slaughter. However, the only significant difference was observed in zone 3, suggesting that the rest period did not influence any parameter including the pHu value. Although no confounding effect related to the rest period was observed, the lower pHu observed in zone 3 is likely attributable to factors specific to that geographic zone, potentially including regional production systems or environmental conditions. These potential confounding effects and other limitations of the dataset are acknowledged in the conclusion.

### 3.2 Variability in carcass data as function of transport time

**Fig. 4:**
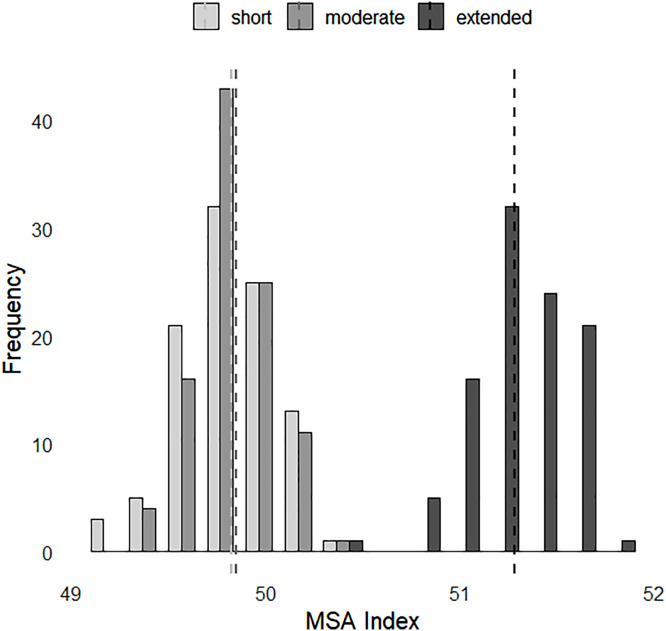
Distribution of MSA Index (*n* = 524 carcasses) across different transport times [short (less than or equal to 22 min), moderate (between 22 and 42 min) and extended (> 42 min)]. The dotted lines represent the average MSA Index for each transport time group. Shades of gray indicate transport duration: light gray for short, medium gray for moderate, and black for extended transport times.

The effects of class of transport time (short, moderate and extended) on beef carcass traits are reported in Table 4 and Figs. 3 and 4.

**Table 4:** Carcass traits in different times divided into short, moderate and extended of the following traits pHu (*n* = 4407 carcasses), Cold Carcass Weight (CCW, *n* = 4407 carcasses), Marbling (*n* = 524 carcasses) and MSA Index (*n* = 524 carcasses).

	Pre-slaughter transport time (h)*			SEM^1^	*P* value
	short	moderate	extended		
pHu	5.77^a^	5.76^a^^,^^b^	5.75^b^	0.002	<0.05
CCW (kg)	408.20^a^^b^	412.21^a^	405.60^b^	1.069	<0.05
Marbling	309.70	296.02	319.33	4.384	NS
Meat Color	2.27	2.36	2.30	0.030	NS

a,b Means with different letters within a row are significantly different (*P* <0.05).

1 SEM - Standard error of the mean.

⁎ The transport time from farms to the slaughterhouse was divided into the following categories for pHu and CCW: short (less than or equal to 47 min), moderate (between 47 and 71 min) and extended (more than 71 min). For MSA data (marbling and color), these categories were short (less than or equal to 22 min), moderate (between 22 and 42 min) and extended (> 42 min).

Mendes et al. (2024b) investigated the effects of stress during transport using a large data set (30,230 Nellore carcasses) and concluded that long distance did not have a significant impact on pHu, and, consequently, on the quality of Nellore beef in Brazil. In their study, animals were sourced within a 300 km radius of the slaughterhouse, corresponding to a maximum transport time of 8 h. In contrast, the present study with Limousin cows in France observed minimal or even negligible effects of distance and transport time on pHu. This is likely due to the low stress levels experienced during transport, supported by the strategy of sourcing animals within a 99 km radius (equivalent to a maximum transport time of 6 h). These findings are consistent with existing literature, which suggests that shorter transport distances and well-managed pre-slaughter conditions can minimize the impact of transport on meat quality (Lacerda et al., 2022; Polkinghorne et al., 2018).

In this study with Limousine cows, transport time—whether short, moderate, or extended—had a significant effect on pHu, CCW, as shown in Table 4, and MSA Index (Fig. 4). However, the effects observed in our study were small. The differences in findings between Mendes et al. (2024b) and our study may be attributed to the distinct physical characteristics of the Nellore breed, as highlighted by Mendes et al. (2024b).

Although we did not directly measure stress indicators (e.g., lactate, glucose, cortisol, creatine kinase, norepinephrine, epinephrine, or bruising scores), our results are consistent with other studies (Gruber et al., 2010; Hultgren, Schiffer, Babol & Berg, 2022; Polkinghorne et al., 2018) that used these indicators to assess the effects of transport on beef quality. These studies, conducted with smaller sample sizes, similarly reported no significant impact of transport time on ultimate pH.

Polkinghorne et al. (2018) found that extending the transport time from 12 to 36 h did not negatively impact beef eating quality according to the MSA grading scheme and physico-chemical analyses. From this, it can be inferred that the present study provided similar results regarding beef eating quality, because transport time was unlikely to be sufficient to cause any significant stress and depletion of glycogen stores in our animals. Longer transport duration may alter the rumen environment, thereby increasing the acetate/propionate ratio, which, in turn, would reduce blood glucose levels and contribute to affect ultimate pH (Deng, He, Zhou, Xu & Xiong, 2017) in addition to glycogen depletion in muscles. However, this is unlikely to have occurred in the animals studied, because even the longest transport time (6 h) did not hinder decline in pHu.

In addition, pHu values were lower for the extended times, which were farther away from the slaughterhouse (5.75 versus 5.77, *P* <0.05, Table 4, Fig. 3), which is possibly due to transport conditions, with driver experience being an important factor. In the development of comprehensive guidelines for reducing cattle transport stress, it is essential to consider multiple factors, including the type or breed of cattle, ambient temperature, transport duration, driver experience, and road conditions (González et al., 2012). In this study, we specifically focused on the impact of transport duration on pHu and meat quality in controlled commercial conditions.

A practical approach to measure stress levels in individual animals at the time of slaughter could involve using threshold values for various indicators, including ultimate pH, within the MSA grading scheme. Such methods for assessing stress would enable more precise evaluations of pre-slaughter practices, such as transport duration and conditions (Polkinghorne et al., 2018).

Transport time, whether short, moderate, or extended, had a significant effect on the MSA Index (Table 4). This effect may be due to the interaction of transport-induced stress with a range of factors contributing to meat quality, such as marbling, hanging method, and ultimate pH (Pethick et al., 2021). In this commercial-scale study, meat color was evaluated as a proxy for ultimate pH, as these measures are generally correlated (Polkinghorne et al., 2018). The absence of any transport effect on color suggests a minimal practical impact of transport time on pHu, reinforcing the finding that transport has a negligible effect on beef quality.

Pre-slaughter transport distance was found to have a slight, but significant effect (Table 4), possibly because other factors, such as animal density, and lot mixing in truck compartments, related to transportation affect carcass weight (Mendonça et al., 2019). Variability in carcass characteristics has multiple sources, and is often related to the priorities of the farming system used (Clinquart et al., 2022). Even when carcasses originate from the same farm with animals of the same gender, there is noticeable variation in carcass characteristics (Chriki et al., 2013; Liu, Ellies-Oury, Stoyanchev & Hocquette, 2022). Along with Bureš and Bartoň (2012) and Silva et al. (2019), we conclude that the age at which an animal is slaughtered determines the weight and composition of the carcass due to the stage of physiological maturity at that time. This supports the result obtained from the variance analysis model.

It is worth noting that improvements in rearing practices at the farm level can be impeded or even completely negated by poor transport, substandard slaughter and processing conditions (Duarte et al., 2011; Mendes et al., 2024b). In the present study, the pre- and post-slaughter protocols used were those recommended by EU legislation and guidelines on Animal Welfare.

Mixed linear models were fitted to data, and confidence intervals for variance components and contrast of least square means were estimated. Results from the 95% confidence interval for the variance of components of the producer effect within different classes of transport time (short, moderate and extended) showed that there is significant variability between producers in Cold Carcass Weight and Marbling, but pHu and MSA Index showed no significant variability between producers (Table 5).

**Table 5:** Lower and upper limits of the 95% confidence interval for the variance components of the producer effect within different times (short, moderate and extended) of the following traits pHu (*n* = 4407 carcasses), Cold Carcass Weight (CCW, *n* = 4407 carcasses), Marbling (*n* = 524 carcasses) and MSA Index (*n* = 524 carcasses).

	σ^2^	
Trait	2.5%	97.5%
pHu	0.00	0.07
Cold Carcass weight	68.09	71.19
Marbling	30.93	60.65
MSA Index	0.00	2.41

Furthermore, the contrasts of least square means revealed statistical significance only between short and extended transport times; regarding ultimate pH (pHu), as well as between moderate and extended transport times for marbling, CCW and MSA Index. The results from the linear mixed model were similar to those from the ANOVA for all carcass traits except for MSA marbling.

## 4 Conclusion

We concluded that neither the distance from the farm to the slaughterhouse nor the transport time to slaughter had little effect on the ultimate pH of beef from Limousin cull cows. Using the MSA Index, potential effects on the eating quality of beef are small, even negligible. However, we observed significant variability in the variables studied, including potential eating quality, both between beef producers within a single zone and between producers in different geographic zones.

Weak effects, or even the absence of effects, of distance and transport time between farm and slaughterhouse prior to slaughter may be explained by low levels of stress experienced by animals during transport, associated with the strategy of only purchasing animals within a radius of 99 km, which is equivalent to a maximum transport time of 6 h.

Although the findings suggest that transport has minimal impact under these conditions, the study did not include physiological stress measurements, such as cortisol, lactate, glucose, or creatine kinase, which could provide deeper insights into the stress levels experienced by animals. Future research could benefit from smaller-scale, controlled studies that incorporate these stress markers to evaluate their effects on beef quality. Such studies would enhance our understanding of how these physiological factors interact with quality monitoring systems along the value chain, ultimately supporting the meat industry's efforts to advance animal welfare and optimize meat quality in commercial pre-slaughter operations.

## Ethics statement

This work did not need any ethical approval since it is based on commercial data already collected by one private company.

## CRediT authorship contribution statement

**Nathalia da Silva Rodrigues Mendes:** Writing – review & editing, Writing – original draft, Formal analysis, Data curation. **Renato Rodrigues Silva:** Writing – review & editing, Writing – original draft, Validation, Formal analysis. **Moïse Kombolo-Ngah:** Formal analysis, Data curation. **Pierre-Philippe Rivet:** Formal analysis, Data curation. **Jerôme Tondusson:** Validation, Funding acquisition. **Tatianne Ferreira de Oliveira:** Writing – review & editing, Validation, Supervision, Funding acquisition. **Sghaier Chriki:** Writing – review & editing, Validation, Supervision. **Marie-Pierre Ellies-Oury:** Writing – review & editing, Validation, Supervision. **Jean-François Hocquette:** Writing – review & editing, Validation, Supervision, Funding acquisition, Conceptualization.

## Declaration of competing interest

The authors declare that they have no known competing financial interests or personal relationships that could have appeared to influence the work reported in this paper.
